# Dental education amid armed conflict in Sudan: Unveiling the impact on training

**DOI:** 10.1371/journal.pone.0311583

**Published:** 2024-10-09

**Authors:** Esra Abdallah Abdalwahed Mahgoub, Samar Osman, Musab Babiker Haga, Amna Khairy, Sarah Hashim Mohammed Osman, Abubker Mohammed Abbu Hassan, Nassifu Ssemwanga, Eiman Gamal Elgaali, Mohamed H. Taha

**Affiliations:** 1 Faculty of Medicine, International University of Africa, Khartoum, Sudan; 2 One Percent Research Initiative, Khartoum, Sudan; 3 Faculty of Dentistry, National University, Khartoum, Sudan; 4 Faculty of Dentistry, National Ribat University, Khartoum, Sudan; 5 Eastern Mediterranean Region Network for Public Health, Khartoum, Sudan; 6 Faculty of Medicine, University of Khartoum, Khartoum, Sudan; 7 Islamic University in Uganda, Mbale, Uganda; 8 College of Medicine and Medical Education Center, University of Sharjah, Sharjah, United Arab Emirates; University of Queensland, AUSTRALIA

## Abstract

The ongoing conflict in Sudan has severely disrupted the health professions education, notably in dentistry. This study aims to explore the impact of the armed conflict on dental education and training. A mixed-method cross-sectional study encompassed 29 dental schools in Khartoum, Darfur, Kordofan States, and Merowe City. Five key informants from the Sudanese Medical Specialization Board, the Human Resource Development Administration, and a university offering clinical dental master’s programs were interviewed. Quantitative data on attacks against dental schools were collected through a structured Google form, and qualitative data on postgraduate training were obtained through semi-structured key informant interviews. Findings reveal that 79.3% of dental schools faced military assaults, with 73.9% experiencing looting and 60.9% repurposed as military bases. Notably, 44.8% of schools shifted to online education,3.4% collaborated with other universities, and 27.6% adopted a combined approach. Key informants’ interviews unveiled disruptions in specialty training and housemanship, limiting access to crucial training facilities. The primary proposed solution was overseas training. Recurrently emphasized strategies to enhance the resilience of the training system included establishing dental centers in all states, collaborating with external training bodies, and anticipating and preparing for potential disasters. The adverse effects of the conflict on both undergraduate and postgraduate dental education are discernible, manifested through resource limitations, a shortage of dental materials and supplies within training facilities, and direct disruptions to clinical training due to attacks on healthcare institutions. The imperative need for urgent interventions is underscored to alleviate these consequences and safeguard the continuity of educational and training efforts.

## Introduction

The conflict in Sudan, which began on April 15, 2023, has shown little sign of subsiding, significantly impacting higher education institutions in Khartoum State and beyond. According to the Sudanese Ministry of Higher Education and Scientific Research, 104 higher education institutions, research centers, and the National Fund for Student Welfare have suffered from sabotage. This widespread disruption extends to all public universities, over ten private universities, two semi-public universities, and twenty university colleges in Khartoum State, all of which have been systematically targeted [1].

Several studies have examined the impact of conflict on higher education. For instance, in Iraq, around 63% of medical schools experienced impairments in the educational process, and 50% reported a decline in the quality of education [2]. Similarly, in Syria, the politicization of health professional education was evident, with attacks on educational institutions causing damage to infrastructure and interruptions in training [3]. The consequences of conflict go beyond physical harm, as in Liberia, the educational process was delayed due to the destruction of teaching and residential facilities, the migration of faculty staff, and a lack of personal security [4]. More recently, the conflict in Ukraine necessitated a shift towards online education, which unfortunately resulted in the suspension of clinical training and formal student assessment [5].

Prior to the conflict, Sudan boasted a robust dental education system, with over 30 colleges offering undergraduate dental programs [6]. Postgraduate programs, mainly provided by the Ministry of Education through the University of Khartoum and the Sudan Medical Specialization Board (SMSB), covered oral surgery, periodontics, pediatric dentistry, oral pathology, and dental public health. However, despite the significant advancements in dental education in the country, the dentistry field in Sudan has been grappling with significant challenges such as inadequate infrastructure, poorly equipped facilities, funding crises, political and economic instability, past civil conflicts, and corruption [7, 8]. These pre-existing challenges further compounded by the reported attacks on educational institutions, damaged infrastructure, faculty shortages, and safety concerns [9, 10].

While the general impact of conflict on education is established, the specific effects on Sudan’s dental education system still need to be clarified. This study aims to investigate the exact effects of the current conflict on dental schools and training programs in Sudan. By addressing this knowledge gap, the research will inform stakeholders in developing strategies to ensure continued access to quality dental education during and after this challenging period.

## Materials and methods

### Study design and population

This is a mixed-method descriptive cross-sectional study that used both quantitative and qualitative methods. The study involved all the dental schools in the areas affected by the ongoing armed conflict in Sudan since April 15, including Khartoum, Darfur, and Kordofan States, in addition to Merowe City. The total number of dental schools in these areas was 29 public, private, and military-related faculties. Additionally, the study involved five key informants from the Board of Oral and Maxillofacial Surgery, the Board of Pediatric Dentistry, the Board of Periodontics in SMSB, the Training Administration in the Human Resource Development Administration of the Federal Ministry of Health, and a university that provides orthodontics master’s degree.

### Study sample

Total coverage of all dental schools in the targeted areas was achieved. Purposive sampling was used to select the key informants based on their experience and knowledge about the concept under study. Five key informants were selected, and the sample size was justified by covering all types of institutions that provide postgraduate clinical dental training in Sudan. Every individual approached willingly agreed to participate in the study without any remuneration.

### Data collection

The quantitative data were collected using a structured Google form composed of 12 questions about the name and type of school, location, attack type, damage extent, and methods used to continue the educational process. Six data collectors documented attacks on universities that occurred between April 15th and July 15th using two main methods: online searches and personal communication. To ensure a systematic approach, the data collectors followed a series of consecutive steps to obtain information about the attacks. If the information available was incomplete or needed further clarification, they would move to the next step.

Step 1: The data collectors first searched for information on the official websites, Facebook pages, and X pages of the universities themselves.

Step 2: Next, they conducted online searches of the Facebook pages and X pages of student associations at the colleges.

Step 3: An extensive online search was then conducted using Google, Facebook, and X. This search utilized both Arabic and English languages and included the following pre-defined keywords: Names of the universities; Attacks; War; Conflict; Looting; Destruction; Sudanese Armed Forces; Rapid Support Forces; SAF; RSF; Sudan; Khartoum; Dental University; Damage; Military Conflicts; Medical Education; Armed Conflict; Dental Schools.

Step 4: Personal communication with college/university staff. It was then conducted with university administrative and academic staff via phone calls or social media applications. The choice of staff to contact was based on their seniority within the administrative hierarchy of the colleges/universities.

Step 5: Finally, personal communication with students or eyewitnesses who had posted information about the attacks on social media.

Throughout the data collection process, data collectors maintained direct contact with E.A.A.M. This ensured they could clarify any questions about the form, the selection of staff to interview, or the choice of eyewitnesses to contact.

For the qualitative data, a semi-structured key informant interview guide was used. Three interviewers, [E.A.A.M, S.O, and M.B.H], conducted the interviews in Arabic. The interviews were conducted by phone and audio-recorded with the participant’s permission. E.A.A.M transcribed all interviews in their original language and then translated them into English. The transcripts were then checked for accuracy by another team member.

### Data analysis procedures

For the quantitative data analysis, data from the form was extracted into an Excel spreadsheet. The data was then analyzed using the Statistical Package for the Social Sciences [SPSS] version 26. The analysis focused solely on descriptive statistics, presented in the form of frequencies and percentages displayed in tables and graphs.

A content thematic analysis of the qualitative data was conducted by N.S. To illustrate the identified themes, unedited participant quotations were included. Each transcript underwent a rigorous analysis process, involving five readings to ensure a deep understanding of the data. Open coding, rather than predefined codes, was employed to allow for the emergence of themes directly from the data. Following the initial coding stage, the codes were systematically organized, compared, and grouped into clear and comprehensive themes and sub-themes that directly addressed the research questions. Finally, the team members discussed and reached consensus on the final themes identified.

### Ethical considerations

Ethical approval for this study was obtained from the International University of Africa, Faculty of Medicine Research Ethical Committee [REC] [Approval number: 06-23/B]. All participants provided verbal informed consent before their enrolment. The consent was obtained through phone calls or social media applications calls, and the primary researcher witnessed it. Before obtaining verbal consent from each participant, the researcher explained the study details, including the purpose, procedures, and the voluntary nature of participation. The researcher ensured that participants could ask questions and clarify any concerns that arose during the consent process. Following this, the participant indicated their consent verbally, and the researcher documented this by making a detailed note in the study records, including the date and time of the verbal consent. The recruitment period for the participants was between the 10th of July and the 7th of August. The participants were permitted to record their interviews, and the anonymity and confidentiality of the participants were ensured.

## Results

### Quantitative findings: Effect of conflict on undergraduate faculties

The mapping process encompassed 29 dental schools, with nearly 50% of the data sourced from the schools’ administrative staff or established connections with current students or faculty. A significant majority, constituting 75%, were private institutions [n = 21], while public schools and those affiliated with the military or police accounted for 20.7% [n = 6] and 6.9% [n = 2], respectively.

Concerning security incidents, a substantial 79.3% of the schools experienced various forms of attacks by military forces, with 73.9% falling victim to looting and 60.9% being repurposed as military bases or utilized for military activities. Notably, all military/police-affiliated schools [n = 2] and 85.7% [n = 18] of private institutions faced attacks during the clashes, compared to half of the public schools mapped [n = 3]. The extent of damages from these attacks remained undetermined for 43.5% of the schools [n = 10]. However, over one-third and approximately one-fifth of the schools reported attacks specifically targeting administrative buildings or essential study sites such as lecture rooms and practicum areas. Further details about these attacks on dental schools are outlined in Table 1.

**Table 1:** Proportion, type, and extent of attacks on dental schools.

			n[%]
School type [n = 29]		Private	21 [72.4%]
		Public	6 [20.7%]
		Related to military forces	2 [6.9%]
Total schools attacked [n = 29]			23 [79.3%]
Attacked schools [n = 23]	Attacks by School-type	Private	18 [78.3%]
		Public	3 [13.0%]
		Related to military forces	2 [8.7%]
	Extent of Structural damage	Lecture halls	2 [8.7%]
		Laboratories	3 [13.0%]
		Administrative buildings	8 [34.8%]
		Student services facilities	2 [8.7%]
		University walls and squares	3 [13.0%]
		No information	10 [43.5%]
	Type of attack	Destruction of the building	9 [39.1%]
		Burning or setting fire	2 [8.7%]
		Looting or stealing	17 [73.9%]
		Conversion into a military base or use for military purposes	14 [60.9%]
Source of information [n = 29]		College/university Staff	24 [82.8%]
		Student/eyewitnesses	15 [51.7%]
		University Facebook/X page	6 [20.7%]
		Student Association Facebook/X page	4 [13.8%]
		Social media posts/comments	12 [41.4%]
		News page	5 [17.2%]
		University Official website	2 [6.9%]
		Single Source*	13 [44.8%]
		Multiple Source	16 [55.2%]

*In 10 out of the 13 colleges, the information was obtained from college/university staff

**Fig 1:**
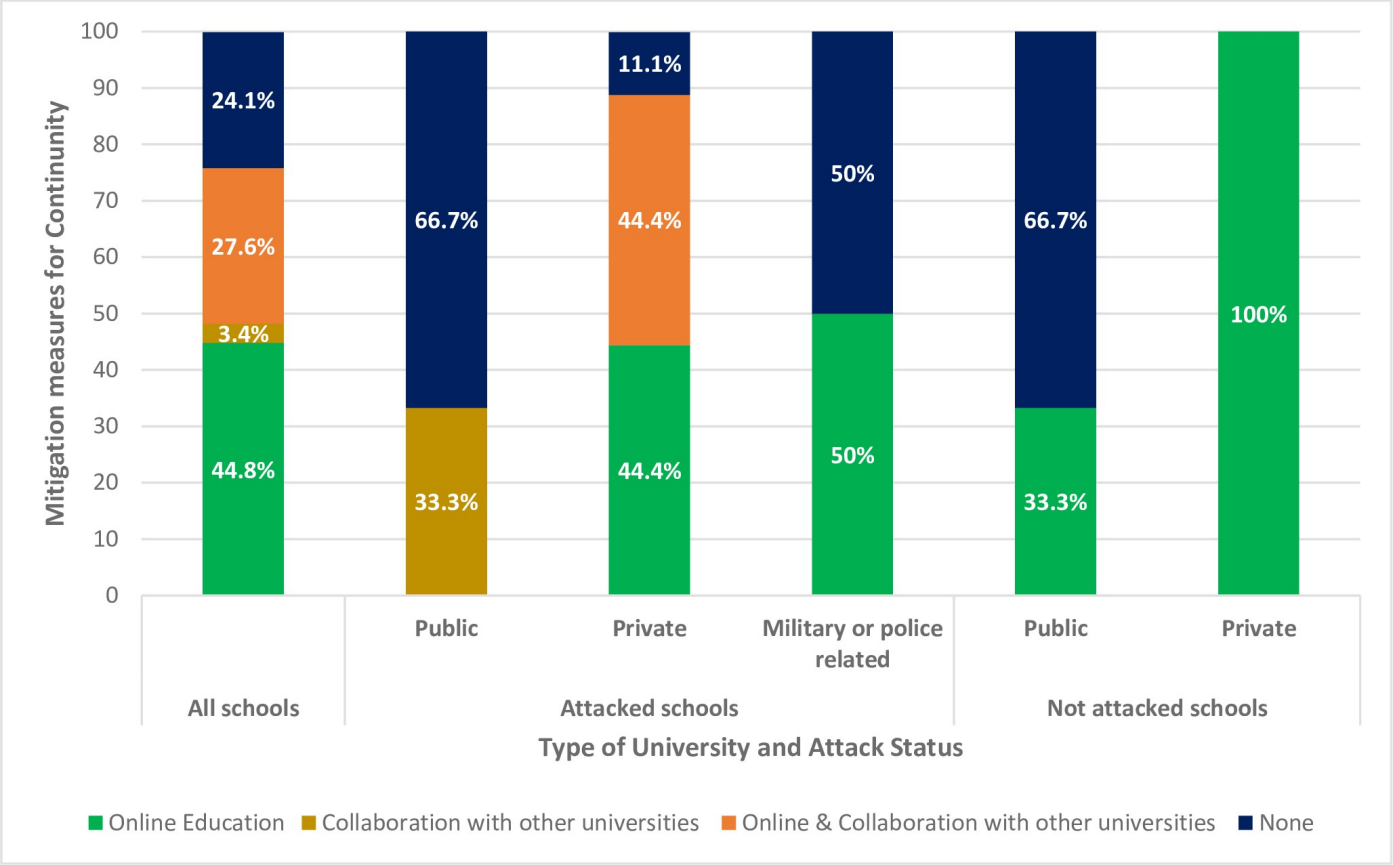
Mitigation measures implemented by universities, classified by type of university (public, private, military or police) and exposure to attack during Sudan armed conflict 2023.

In response to the potential prolonged suspension of the academic year, universities implemented various measures to mitigate risks. These strategies included transitioning to online learning [44.8%, n = 13], collaborating with other universities [3.4%, n = 1], or employing a combination of both measures [27.6%, n = 8]. Collaborative efforts involved partnerships with either national or African international universities. The study revealed that seven colleges failed to implement a substantive solution to maintain the educational process. Among the attacked institutions, 88.8% [n = 16] of private schools implemented mitigation measures [online learning and/or collaborating with other universities], while 33.3% [n = 1] of public universities and 50% [n = 1] of military-affiliated schools applied similar strategies [Fig 1].

### Qualitative findings: Effect of conflict on postgraduate training

To provide context for the findings, each participant is succinctly described, utilizing pseudonyms for Key Informant Interview [KII] participants. S1 Table offers a concise overview of the profiles of the KII participants, delineating their designations, affiliated institutions and fields of experience.

**Fig 2:**
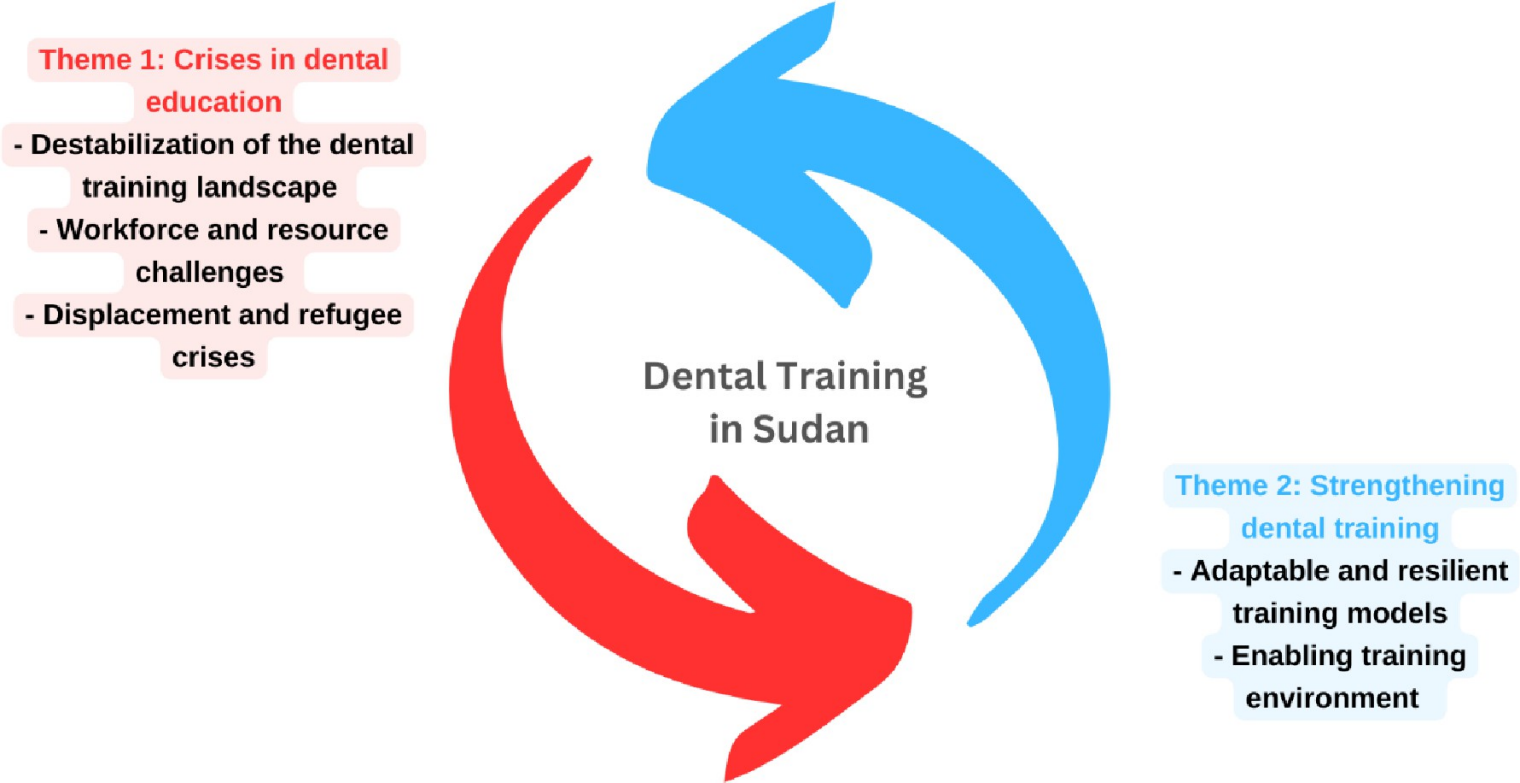
Illustration of the key themes and sub-themes.

Two themes emerged from the information unravelling including “Crises in Medical Education” and “Strengthening Dental Training”. These themes are further presented through distinct sub-themes and categories, providing a comprehensive exploration of the findings. Exacts from the transcripts have been presented in italics to support the findings [Fig 2, S2 and S3 Tables].

#### Theme 1: Crises in dental education

This theme presents the findings regarding the effect of the conflict on the training of dentists in Sudan. It comprises three sub-themes including destabilization of the medical training landscape, workforce and resource challenges and displacement and refugee crises. The participants’ insights offer nuanced perspectives on the multifaceted impact of the conflict on dental training.

*Sub-theme 1*: *Destabilization of the dental training landscape*. The participants highlighted a myriad of effects on the medical training landscape including deferred internship placements, disruption of training programs, disjointed communication and connectivity and disruption of medical credentialing and certificates. The destabilization of the training environment is vividly portrayed through participants’ narratives.

Several participants noted that the war had a significant impact on the internship placement program for dental students, resulting in some placements being postponed and others not being assigned at all.

“…*the August 2020 list of doctors was distributed in November*” KI-1

“…*the list of 2021 who were expected to start their internship in May was not distributed*” KI-1

“*Those who are abroad were not distributed…*” KI-4

The majority of participants expressed that the war led to disruptions in dental training programs, with many clinical training activities coming to a halt.

“*The training stopped*” KI-4

“*The program stopped*” KI-5

“…*training stopped entirely*” KI-1

“…*stop the problem of clinical training until the situation calmed down*” KI-3

“*Direct training stopped completely*” KI-3

Participants highlighted that communication became challenging due to the unrest in Sudan, impacting both in-person and online education.

“…*difficult to communicate*” KI- 4

“…*we can’t communicate*” KI-3

They also noted connectivity and internet issues hindering online training opportunities.

“*The opportunity to work online is a problem*” KI-5

“*10% had no internet access*” KI-3

Furthermore, some participants pointed out disruptions in the medical credentialing and certification process due to the war.

“…*the war affected the issuance of certificates*” KI-2

“…*practicing the job without a license is impossible*” KI-3

*Sub-theme 2*: *Workforce and resource challenges*. This sub-theme highlights the participants perspectives on the scarcity of medical specialists and trainers, inadequate training facilities, and remuneration disparities. Their accounts shed light on the systemic challenges.

The participants voiced concerns regarding a dearth of medical specialists and trainers, particularly noticeable in the states.

“…*absence of specialists*” KI-1

“*…there are no supervisors in the states and their number is small*” KI-5

“…*distributing specialists in the States was impossible*” KI-1

Moreover, participants highlighted a scarcity of training facilities, noting prolonged closures due to the conflict.

“*…lack of training facilities*” KI-1

“*…some centers closed for a long time*” KI-1

“…*no place for training except for a fee*” KI-4

This shortage of resources extended to the state health centres. The participants highlighted that the available facilities are not well prepared to support training of dentists.

“…*health centers in states were unprepared and unequipped*” KI-1

“…*they could go to an entire state without finding a single dental chair in it*” KI-1

“…*hospitals in Sudan are not prepared*” KI-3

KI-1 emphasized that the specialists in the hospitals are also poorly renumerated with many working on a voluntary basis.

“…*a minimal amount of money*” KI-1

“…*work remained entirely voluntary*” KI-1

*Sub-theme 3*: *Displacement and refugee crises*. Participants shed light on the impact of the conflict on dental training institutions and personnel, particularly through incidents of attacks on universities and the consequent displacement of students and specialists. The participants indicated that some training institutions were attached during the war causing a refugee crisis with many students and specialists displaced.

“*The university was attacked*” KI-5

“*60% of the candidates were in Egypt*” KI-3

“*Some specialists went outside Sudan*” KI-1

#### Theme 2: Strengthening dental training

This theme highlights the proposed interventions to mitigate the adverse effects of unrest on dental education. It comprises two sub-themes, "Adaptable and Resilient Training Models" and "Enabling Training Environment," this theme encapsulates participants’ suggestions for fostering resilience and continuity in dental education amidst challenging circumstances.

*Sub-theme 1*: *Adaptable and resilient training models*. Participants advocate for decentralized training approaches, cross-border collaborations, and digital transformation as strategies to ensure the continuity of dental education during times of crisis. They emphasized that these interventions should be implemented taking into account the number of specialists and trainers in various states.

“*The training should not be confined to the capital*” KI-4

“*State centers should be part of the routine to ensure continuity of training*” KI-4

“*…distribute intern doctors within the state according to hospitals and according to the presence of specialists in the hospital*” KI-1

Some participants recommended incentivizing trainers and specialists to relocate to the states to ensure continuous training of dentists. KI-3 proposed engaging with state authorities to facilitate doctor training.

“…*communicate with the authorities in the states to train the doctors*” KI-3

“*Count the candidates and their geographical locations and see if there is a trainer in these places*” KI-3

Furthermore, some participants continued training dentists in states away from the affected capital Khartoum, as KI-4 noted.

“*…registrars inside Sudan, they were distributed in government hospitals*” KI-4

“*…doctors are distributed according to their state and what is available in the hospitals*” KI-1

Cross-border collaboration with training institutions and licensing bodies in other countries was suggested to address challenges arising from the conflict in Sudan.

“*The council should have external cooperation and twinning with some universities or hospitals abroad*” KI-4

“*…cooperating with universities outside the country*”KI-5

“…*training should be in partnership with another country*” KI-3

“…*students can study the program the same way in other institutions inside or outside the country*” KI-5

Some participants expressed that digital transformation is crucial for ensuring training continuity, citing online education and certification.

“*…advance the electronic system …replaced by electronic certificates…*” KI- 2

“*…continue online seminars*” KI-3

“*…opportunity to work online*” KI-5

“…*we need stronger and effectiveness electronic*, *server clouds and backup for our database*” KI-2

“*WhatsApp groups were created for easy communication*” KI-4

The participants emphasized that there is a need for adaptive strategies to continue training dentists amidst the war crisis. For instance, some participants highlighted that they could pause clinical training and concentrate on theoretical modules such as research.

“*…we changed the plan to address the thesis issue*” KI-3

“…*the first year of training should be dedicated to research and online education*, *and the second and third years to clinical training*” KI-5

“…*map the dental centers and at least try on the* light shifts” KI- 1

*Sub-theme 2*: *Enabling training environment*. Participants stress the need for supportive policies, infrastructure development, and incentives to attract and retain dental professionals in underserved regions. Their insights highlight the pivotal role of creating an enabling environment to sustain the continued training of dentists. This includes capacity building and ensuring fair renumeration and incentivization for trainers.

“*…the government should care about state hospitals and state training centers*” KI-2

“*…attention of the workforce…*” KI-1

“…*open new dental centers*” KI- 1

Furthermore, participants underscored the necessity of fostering an environment that encourages specialists and dentists to establish practices in the states.

“*…encourage and provide the appropriate environment for trainees and trainers to work in the states*” KI-2

“*…many dentists have begun to open dental centers in the states*” KI-1

“…*budgets should increase to an extent sufficient for the needs of state training centers*” KI-2

Some participants highlighted the importance of fairly compensating specialists and trainers who choose to work in underserved regions. KI-1 recommended increasing their income to incentivize their relocation and retention.

“*…increase their income*” KI-1

## Discussion

This descriptive cross-sectional study was conducted among dental schools in armed conflict-affected areas, along with key informants from postgraduate clinical dental training in Sudan. The study unveiled that approximately 80% of the schools experienced various forms of attacks by military forces. The postgraduate training was also significantly disrupted, and efforts with constrained capacity were undertaken to sustain the training of dentists.

According to the current study, a significant majority of dental schools in Sudan [79.3%] have experienced different types of attacks, similar to what has been observed in conflict areas. Studies conducted in countries like Syria, Iraq, Ukraine, Palestine, and Liberia have similarly documented extensive damage to the infrastructure of the health professional education institutions [such as university libraries and laboratories] and the exposure of these institutions to various forms of attacks [2–4, 11–15]. Our findings underscore the immediate necessity to protect educational institutions by advocating for the enforcement of international humanitarian and criminal laws denouncing assaults on educational facilities [16] and ensuring that the international community holds these actions accountable. The occurrence of these attacks resulted in the suspension of dental training, and research has indicated that such disruption in training can adversely affect the health of the population [17, 18].

More than one-third of the faculties in our study suffered damage to administration offices. Attacks on these important facilities may threaten to retrieve the student’s data and academic records. The loss of such data may exacerbate the difficulties for students and impede their ability to pursue further education in universities in secure regions or overseas. Therefore, transformation to digital documentation and cloud backup plans should be considered by faculties.

Moreover, the war in Sudan has resulted in a substantial financial burden for dental schools in the country, with 73.9% of surveyed schools experiencing looting or theft. This situation further compounds the resource challenges many dentistry schools face in Sudan due to the country’s economic environment [19]. Given the significant financial demands placed on faculty for physical resources required for dentistry programs, such as dental materials, clinic devices, and units, the repercussions of losses incurred during this war will likely impede their future recovery. According to a study by Stafford et al., dental education expenses have been rising faster than the actual net income of dentists in practice over the past decade [20].

The study revealed that a majority of dental schools [60.9%] reported the use of dental campuses either for military purposes or as military bases. This trend aligns with transforming university campuses into military bases in other wars across the globe, such as in Afghanistan, Sierra Leone, and Libya [15]. Our study revealed that the two military-affiliated dental schools in Khartoum state have been repurposed as military bases. This raises concerns about the feasibility and safety of establishing medical colleges associated with the military or police in politically unstable countries. Such affiliations pose a risk of potential danger to these institutions during armed conflict, an issue currently being observed.

Following the disruption of the educational system due to the expansion of armed conflict zones, the prevailing solution to restore the learning process among the studied dental schools was the transition to online education. A similar experience was reported in conflict-affected countries like Ukraine and Liberia [21, 22]. However, implementing this approach in conflict areas can be challenging due to various obstacles, including unstable network connections, limited bandwidth, frequent power outages, and the absence of IT support [21, 22]. All these barriers are also evident in Sudan post-war. Consequently, utilizing asynchronous online learning [10] and leveraging popular social media platforms like Twitter [23], Telegram [24], Facebook [25] and YouTube [26] could serve as cost-effective alternatives to establishing dedicated educational platforms.

Our study found that around one-third of the dental schools have established collaboration with universities in the safe states. By sharing the resources with the universities in the safe states in the country, it is possible to guarantee education in a safer setting and optimize the utilization of available resources [10, 27]. Nevertheless, the expansion of the conflict in mid-December to other areas, such as Gezira State [28] which accommodated several relocated universities and acted as the administrative center for the SMSB, has the potential to raise doubts about the effectiveness of this solution within the Sudanese context. It is worth noting that transitioning into e-learning and collaboration with other universities were among the measures initiated by 90% of private schools against the risk of long suspension of the academic year. However, only half or less of the military and public schools adopted this measure. This indicates that, regardless of the national circumstances, private schools are more devoted to fulfilling the education cycle and financial commitments.

Our research findings indicate that the impact of the war on housemanship and clinical specialty training was profound. Initially, both were postponed, but efforts were made to resume the training gradually. Similar interruption in clinical training due to armed conflict was documented in other countries like Syria [3], Iraq [29], Liberia [4] and Ukraine [30].

In Sudan, the interruption of training can be elucidated by three primary factors. Firstly, there is a concentration of resources. Secondly, the dental clinical training in Sudan was already grappling with a scarcity of dental materials and supplies within its training facilities. The third aspect is the impact of attacks on healthcare institutions, directly affecting clinical training.

The first factor is the concentration of resources, such as clinical training facilities and trainers, in the capital state of Khartoum, which is the primary site of the current conflict. For decades, the health system in Sudan has been suffering from uneven distribution of health services in different states [31]. The 2021 annual report of the Ministry of Health highlighted the discrepancy in the number of dentists and dental services between the states [32]. Khartoum state alone accounted for 70.5% of the available dental beds in the country and has the highest density of dentists per 100,000 population among the states [32]. This indicates that serious efforts are needed for the fair distribution of health services and facilities, thus encouraging the training environment for trainees and specialists in all states other than the capital.

Secondly, dental clinical training in Sudan has already suffered from a shortage of dental materials and supplies in the training facilities. Most of these are imported from other countries, mainly India, Belgium, and the Netherlands [33, 34]. The availability of materials will further decrease due to the restricted export movement due to the war and the ongoing inflation that has persisted since then. This indicates that distributing trainees to war-free states may not be feasible at this stage, as most of the safe states lack adequate training infrastructure, with many health centers ill-prepared and lacking necessary materials and equipment, as well as the shortage of specialists in various regions.

The third factor is that clinical training was affected by the attacks on healthcare institutions. Since the first week of the conflict, Khartoum Teaching Dental Hospital, the leading dental teaching hospital in the capital, was attacked and forcefully evacuated. Since then, attacks on healthcare facilities continued, resulting in more than 80% of hospitals in Sudan being out of service [35, 36]. As the reconstruction of the healthcare infrastructure following a conflict is a protracted endeavor, the destruction of teaching hospitals and clinics may be identified as the primary impediment to reestablishing specialized medical training in the country.

Training disruption could exacerbate a pre-existing shortage of dental specialists and limited opportunities for dental specialty training. According to the Sudanese Medical Council’s latest records, there were only 519 registered dental specialists, a relatively small number compared to the 8,492 registered general dental practitioners [33]. With the expansion of the war into new regions and the growing number of displaced individuals abroad, it is imperative for the SMSB to assert its responsibility in offering resolutions for its trainees abroad. This involves collaborating with overseas training institutions and utilizing diaspora initiatives to provide e-learning, mentorship, and exchange opportunities for trainees outside Sudan [10].

Before the war, the housemanship program for dentists in Sudan has long faced challenges. This included limited hospital training opportunities and a lack of necessary materials for training [33]. These issues prompted the formation of the "Pre-Intern Gathering of Sudanese Dentists" to advocate for their rights to training and expedite the training process for doctors on waiting lists. It is anticipated that the number of doctors on the waiting list will increase as a result of the war, given the impact on many training centers. The Human Resources Development [HRD] training administration should develop a long-term plan to address this issue and evaluate the feasibility and cost of expanding training to additional states.

Great efforts are needed to overcome the current challenges facing housemanship and clinical specialty training. The establishment of coordination and collaboration between the HRD’s Training Administration, which is responsible for house officers, and the SMSB, which is responsible for specialists and clinical specialty training, can decrease the duplication of efforts and allow the sharing of data regarding counting specialists and their distribution in war-free states. Accordingly, house officers and specialty trainees could be distributed to states with minimally acceptable essential resources.

### Study limitations

The ongoing conflict in Sudan significantly hampered our data collection efforts. Security concerns prevented direct assessment of dental schools, necessitating reliance on the available data sources. This approach, however, may have introduced inaccuracies due to potentially incomplete or outdated information, as well as the personal biases of the sources. Our qualitative study, which involved interviews with key informants also faced limitations. The sample size might not have been large enough to capture the full range of relevant themes. Additionally, focusing on high-level informants could have introduced selection bias, potentially overlooking the broader experiences of other staff and students. Furthermore, widespread displacement and disruptions to electricity and network infrastructure made it difficult to reach some targeted participants. We acknowledge the potential for bias stemming from our own backgrounds and perspectives to influence data collection and analysis. While efforts were made to mitigate these biases through reflexivity and data triangulation, they remain a consideration. While acknowledging the limitations of our study, we believe it offers valuable insights into the war’s effects on dental education in Sudan.

## Conclusion

The study underscored the adverse effects of armed conflict on dental education, aggravated by looting and facility damage that compounded existing resource constraints. Dental schools responded by adopting e-learning and partnering with national and international universities to avert the risk of academic disruptions. The impact of the war extended to housemanship and clinical specialty training, prompting gradual resumption efforts. Nevertheless, challenges persisted due to resource concentration in Khartoum, dental material shortages, and attacks on healthcare institutions. Addressing these challenges necessitates coordinated local and international efforts to redistribute trainees and reinstate clinical specialty programs. Recommendations to enhance the system and proactively address future crises include establishing fully-equipped dental centers in every state, collaborating with certified training facilities, integrating online education, and fortifying electronic systems for robust data storage and accessibility. Collectively, these comprehensive strategies aim to foster a sustainable training environment and bolster the resilience of dental education in Sudan.

## Supporting information

S1 Table Brief profile of key informant interview participants. (DOCX)

S2 Table Analysis of key informant’s responses about the effect of the war on dental postgraduate training. (DOCX)

S3 Table Emergent themes from key informant interviews. (DOCX)
