# Supplementary material for: Dental education amid armed conflict in Sudan: Unveiling the impact on training
Source: PLoS One. 2024 Oct 9;19(10):e0311583. doi: 10.1371/journal.pone.0311583 (PMC11463757; doi:10.1371/journal.pone.0311583)
Supplement: S1 Table — (DOCX) [file pone.0311583.s001.docx]

**S1 Table. Brief profile of Key Informant Interview participants**

| **Pseudonym** | **Designation** | **Institution** | **Experience** |
| --- | --- | --- | --- |
| KI – 1 | Dental Internship Program Representative | Directorate of Human Resources Development- Federal Ministry of Health | A dentist holding a senior leadership position in dental internship program. |
| KI – 2 | Board Member, Oral and Maxillofacial Surgery Council | Sudan Medical Specialization Board | An Assistant Professor of Maxillofacial Surgery at a reputable public university. He holds a leadership position as the Oral and Maxillofacial Surgery Council, and possess over 10 years of experience in academic work. |
| KI – 3 | Board Member, Paediatric Dentistry Council | Sudan Medical Specialization Board | A paediatric dentist consultant with specialization in Professional Education holds leadership positions shaping educational and training standards. Her involvement includes the Paediatric Dentistry Specialization Council in addition to more than 15 years of experience as a lecturer at universities. |
| KI – 4 | Board Member, Periodontic Council | Sudan Medical Specialization Board | An Assistant Professor of Periodontology. They hold a prominent leadership role within the Sudan Medical Specialization Board's Periodontic Council, demonstrating their expertise and influence in shaping the field. |
| KI – 5 | Private University Representative* | The Private University | An orthodontist who lectures at private universities and he is a member of the Sudanese Orthodontic Association. |

*The name of the university was not mentioned to ensure confidentiality
