# Supplementary material for: Dental education amid armed conflict in Sudan: Unveiling the impact on training
Source: PLoS One. 2024 Oct 9;19(10):e0311583. doi: 10.1371/journal.pone.0311583 (PMC11463757; doi:10.1371/journal.pone.0311583)
Supplement: S2 Table — (DOCX) [file pone.0311583.s002.docx]

**S2 Table. Analysis of key informant’s responses about the effect of the war on dental postgraduate training**

| **Category** | **Sub-theme** | **Theme** |
| --- | --- | --- |
| Deferred internship placement | Destabilization of the dental training landscape | Crises in dental education |
| Disruption of dental training programs |  |  |
| Disjointed communication and connectivity |  |  |
| Disruption of medical credentialing and certification |  |  |
| Shortage of dental specialists and trainers | Workforce and resource challenges |  |
| Absence of facilities |  |  |
| Facility readiness to train |  |  |
| Unsatisfactory renumeration |  |  |
| Displacement | Displacement and refugee crises |  |
| Institutional instability and attacks |  |  |
| Decentralized dental training amid resource constraints | Adaptable and resilient training models | Strengthening dental training |
| Cross-border collaboration |  |  |
| Digital transformation of dental training |  |  |
| Adaptive strategies to continue training |  |  |
| Capacity building | Enabling training environment |  |
| Fair renumeration and incentivization of trainers |  |  |
