# Supplementary material for: Dental education amid armed conflict in Sudan: Unveiling the impact on training
Source: PLoS One. 2024 Oct 9;19(10):e0311583. doi: 10.1371/journal.pone.0311583 (PMC11463757; doi:10.1371/journal.pone.0311583)
Supplement: S3 Table — (DOCX) [file pone.0311583.s003.docx]

| **S3 Table. Emergent Themes from Key Informant Interviews** | | | | |
| --- | --- | --- | --- | --- |
| ***Participants (pseudonyms)*** | ***Unit of meaning = code*** | ***Category*** | ***Sub-theme*** | ***Theme*** |
| ***EFFECTS OF THE WAR ON THE TRAINING*** | | |  |  |
| **KI-1** | No distribution of the interns | Deferred internship placement | Destabilization of the Dental Training Landscape | Crises in Dental Education |
| **KI-1** | The August 2020 list of doctors was distributed in November |  |  |  |
| **KI-1** | The list of 2021 who were expected to start their internship in May was not distributed |  |  |  |
| **KI-4** | Those who are abroad were not distributed |  |  |  |
| **KI-1** | Doctors on the 2021 list have been postponed |  |  |  |
| **KI-2** | Disrupting training | Disruption of Medical Training Programs |  |  |
| **KI-4** | The training stopped |  |  |  |
| **KI-5** | The program stopped |  |  |  |
| **KI-1** | Training stopped entirely |  |  |  |
| **KI-2** | The war stopped the training centers |  |  |  |
| **KI-3** | Direct training stopped completely |  |  |  |
| **KI-4** | How do they sit for the exam |  |  |  |
| **KI-3** | Stop the problem of clinical training until the situation calmed down |  |  |  |
| **KI-4** | Difficult to communicate | Disjointed Communication and Connectivity |  |  |
| **KI-3** | 10% had no internet access |  |  |  |
| **KI-3** | Not all trainees responded, |  |  |  |
| **KI-3** | We can't communicate |  |  |  |
| **KI-5** | The opportunity to work online is a problem |  |  |  |
| **KI-3** | The second thing that is considered an obstacle is the data. We have a problem in that there needs to be networking to ease data access. |  |  |  |
| **KI-5** | Opportunity to work online is challenging |  |  |  |
| **KI-2** | The war affected the issuance of certificates | Disruption of Medical Credentialing and Certification |  |  |
| **KI-2** | The war affected the issuance of certificates |  |  |  |
| **KI-3** | The first obstacle is obtaining a license to practice in the country |  |  |  |
| **KI-3** | You cannot practice the profession |  |  |  |
| **KI-3** | Practicing the job without a license is impossible |  |  |  |
| **KI-1** | Presence of all specialists | Shortage of Dental Specialists and Trainers | Workforce and resource challenges |  |
| **KI-1** | Absence of specialists. |  |  |  |
| **KI-5** | There are no supervisors in the states and their number is small |  |  |  |
| **KI-3** | In addition to 5 trainers representing 50% of trainers |  |  |  |
| **KI-3** | We have yet to find trainers for students in Sudan |  |  |  |
| **KI-1** | Distributing specialists in the States was impossible |  |  |  |
| **KI-1** | Lack of training facilities | Absence of facilities |  |  |
| **KI-1** | Some centres closed for a long time |  |  |  |
| **KI-4** | No place for training except for a fee |  |  |  |
| **KI-1** | Health centres in states were unprepared and unequipped | Facility readiness to train |  |  |
| **KI-1** | No material |  |  |  |
| **KI-1** | They could go to an entire state without finding a single dental chair in it |  |  |  |
| **KI-3** | Hospitals in Sudan are not prepared |  |  |  |
| **KI-3** | Hospitals don’t have a sperate department of paediatrics dentistry |  |  |  |
| **KI-1** | Most of the centres stopped insurance |  |  |  |
| **KI-1** | A minimal amount of money | Unsatisfactory renumeration |  |  |
| **KI-1** | Work remained entirely voluntary |  |  |  |
| **KI-3** | 60% of the candidates were in Egypt | Displacement | Displacement and Refugee crises |  |
| **KI-1** | Some specialists went outside Sudan |  |  |  |
| **KI-5** | The university was attacked | Institutional Instability and attacks |  |  |
| **PROPOSED SOLUTIONS** | | | | |
| **KI-1** | Distribute intern doctors within the state according to hospitals and according to the presence of specialists in the hospital | Decentralized Dental Training Amid Resource Constraints | Adaptable and Resilient training models | Strengthening Dental Training |
| **KI-1** | Doctors are distributed according to their state and what is available in the hospitals. |  |  |  |
| **KI-1** | Prioritized the 2020 doctor’s list |  |  |  |
| **KI-2** | Emigration of trainers from Khartoum to the states |  |  |  |
| **KI-3** | Communicate with the authorities in the states to train the doctors |  |  |  |
| **KI-4** | Registrars inside Sudan, they were distributed in government hospitals |  |  |  |
| **KI-2** | Join the nearest training unit |  |  |  |
| **KI-2** | Supported the training centres in the states |  |  |  |
| **KI-4** | Registrars outside and inside Sudan and their areas of presence were actually counted, as well as the periodontic specialists |  |  |  |
| **KI-3** | Count the candidates and their geographical locations and see if there is a trainer in these places |  |  |  |
| **KI-1** | They should be distributed in the States |  |  |  |
| **KI-4** | The training should not be confined to the capital |  |  |  |
| **KI-1** | Even if they did not settle in this state and limit their work to a period of three to four days a week |  |  |  |
| **KI-4** | State centers should be part of the routine to ensure continuity of training |  |  |  |
| **KI-1** | We contacted the Medical Council to approve the shifts they completed in Sudan | Cross-Border Collaboration |  |  |
| **KI-1** | To do the remaining shifts outside |  |  |  |
| **KI-2** | Sudanese doctors working abroad |  |  |  |
| **KI-5** | Contact universities in Egypt with four supervisors |  |  |  |
| **KI-3** | The Sudanese Medical Council addressing the government agencies to facilitate the registration of Sudanese doctors |  |  |  |
| **KI-2** | Addressed all the training bodies outside Sudan |  |  |  |
| **KI-2** | Oversee the training of trainees who migrated to them outside Sudan |  |  |  |
| **KI-2** | Provide materials from abroad |  |  |  |
| **KI-5** | Cooperating with universities outside the country |  |  |  |
| **KI-5** | Twinning with universities outside Sudan |  |  |  |
| **KI-3** | Training should be in partnership with another country |  |  |  |
| **KI-3** | Continue training anywhere |  |  |  |
| **KI-4** | The council should have external cooperation and twinning with some universities or hospitals abroad |  |  |  |
| **KI-5** | There is no standard program so that students can study the program the same way in other institutions inside or outside the country |  |  |  |
| **KI-2** | Replaced by electronic certificates | Digital Transformation of Dental Training |  |  |
| **KI-3** | Continue online seminars |  |  |  |
| **KI-5** | Opportunity to work online |  |  |  |
| **KI-4** | WhatsApp groups were created for easy communication |  |  |  |
| **KI-1** | No-objection letters online |  |  |  |
| **KI-5** | Online education |  |  |  |
| **KI-2** | Advance the electronic system |  |  |  |
| **KI-2** | We need stronger and effectiveness electronic, server clouds and backup for our database |  |  |  |
| **KI-3** | The data for any program should be saved in two places, so if there is any problem in one place, it is in the backup |  |  |  |
| **KI-2** | Supervise the thesis level, | Adaptive Strategies to Continue Training |  |  |
| **KI-3** | We changed the plan to address the thesis issue |  |  |  |
| **KI-2** | Revive training activities from the discussion forum and theories |  |  |  |
| **KI-1** | Map the dental centres and at least try on the light shifts |  |  |  |
| **KI-5** | The first year of training should be dedicated to research and online education, and the second and third years to clinical training |  |  |  |
| **KI-1** | Attention of the workforce and the investment force | Capacity Building | Enabling training environment |  |
| **KI-1** | Open new dental centers |  |  |  |
| **KI-1** | Many dentists have begun to open dental centers in the states |  |  |  |
| **KI-1** | Specialists who are thinking of having a center in the states to work |  |  |  |
| **KI-2** | Level of service provided in the states |  |  |  |
| **KI-2** | The government should care about state hospitals and state training centers |  |  |  |
| **KI-2** | Budgets should increase to an extent sufficient for the need of state training centers |  |  |  |
| **KI-1** | Increase their income | Fair renumeration and incentivization of trainers |  |  |
| **KI-2** | Encourage and provide the appropriate environment for trainees and trainers to work in the states |  |  |  |
